# Application of lentil seed extract as an inhibitor to assess the corrosion properties of copper–nickel alloys in a NaCl environment†

**DOI:** 10.1039/d4ra04758c

**Published:** 2024-09-03

**Authors:** Mona M. Nageeb, Ghalia A. Gaber, Amal S. I. Ahmed, Abd El Aziz S. Fouda

**Affiliations:** a Department of Chemistry, Faculty of Science (Girls), Al-Azhar University Nasr City Cairo Egypt; b Department of Chemistry, Faculty of Science, Mansoura University Mansoura-35516 Egypt asfouda@mans.edu.eg +2 050 2365730

## Abstract

Weight loss (WL), electrochemical, and surface analysis were used to explore the efficiency of lentil seed extract (LSE) in mitigating the electrochemical corrosion of Cu–10Ni and Cu–30Ni alloys in obviously aerated water. The adsorption of lentil seed extract (LSE) species to create a barrier layer improved the corrosion resistance of Cu–Ni alloys in a NaCl medium. It was shown that the temperature of the medium and the amount of extract used affected the enhanced inhibitory efficacy. Using the PDP approach, the Cu–10Ni alloy showed the maximum inhibition performance (IE) of about 98.58% and 8.53% with 300 ppm LSE, respectively. According to the findings, the studied extract had a good ability to slow down the step at which alloys corroded in a 3.5% NaCl solution. It was discovered that as the temperature rose, the rate of corrosion increased. The thermodynamic activation functions of the dissolution process were also calculated as a function of extract dose. PDP curve analysis reveals that LSE is a mixed-type inhibitor, and EIS findings demonstrates that increasing dose not only alters the charge transfer (*R*_ct_) of Cu–10Ni alloy from 1031 to 2984 Ω cm^2^ and for Cu–N30Ni alloy from 3093–6208 Ω cm^2^ but also changes the capacitance of the adsorbed double layer (*C*_dl_) for Cu–10Ni alloy from 728–678 μF cm^2^ and for Cu–30 Ni alloy from 726 to 701 μF cm^2^. The inhibitor's adsorption provides a good fit for the “Freundlich, Temkin, and Langmuir isotherm” models. Several methods are used to confirm that the alloy surface has a protective coating.

## Abstract 2

Weight loss (WL), was used to explore the efficiency of lentil seed extract (LSE) in mitigating the corrosion of Cu–10Ni and Cu–30Ni alloys in obviously aerated water.

## Graphical abstract

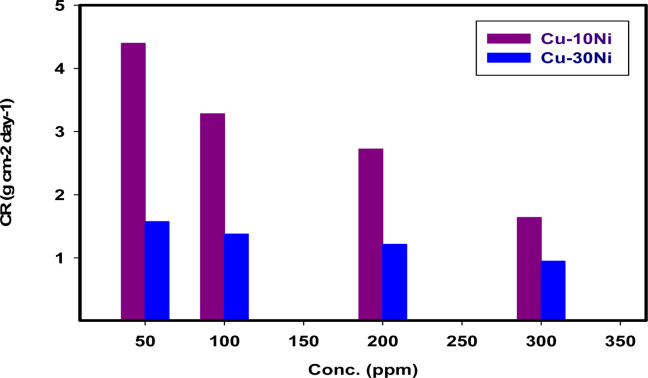

## 1. Introduction

For many years, cupronickel has been extensively utilized as a building material for desalination plants, power plants, ships, heat exchangers and condensers, and seawater pipelines due to its exceptional resistance to corrosion.^1–4^ The protective layer that primarily consists of an inner barrier layer with noticeable amounts of oxygen, copper, and some nickel as well as a thick outer layer primarily composed of cuprous chloride is responsible for the corrosion opposition of Cu–Ni alloys. The alloy has outstanding resistance to corrosion because of its inner layer.^5^ However, after a prolonged exposure period, significant corrosion invariably manifests itself in either hydrodynamic or calm conditions.^6^ Thus, enough attention has been paid to the preservation of copper and copper–nickel alloys, particularly in saltwater environments.^7–9^ The economy is greatly impacted by corrosion, which affects “infrastructure, transportation, utilities, production, and manufacturing”. For this reason, a lot of research is being done to find effective methods and actions to protect metals and composites from corrosion in all types of industrial settings.^10–13^ In order to prevent corrosion on metals and alloys, organic compound-based corrosion inhibitors are frequently employed.

The majority of these substances, however, are synthetic chemicals that can be very costly, poisonous, and dangerous for the environment and living things. As a result, one of the essential procedures that should be modified to safeguard metals and composites from damage in a variety of industrial settings is the employment of environmentally friendly inhibitors. In order to achieve this, plant extracts have recently been evaluated as corrosion inhibitors in various media; encouraging results have been published.^14–19^ These extracts are entirely soluble in aqueous media, nontoxic, and reasonably priced. One of the most significant metallic corrosion inhibitors is plant extract. They are renewable, easily accessible, harmless, safe for the environment, biodegradable, and very effective. The various plant sections that can be extracted are significant since they contain a wealth of chemical compounds.^20,21^ These compounds can be easily and affordably extracted using simple techniques. As a result, studies on plants that naturally inhibit corrosion have drawn interest and are highly important from both a scientific and practical perspective.^22–27^ Chieb, *et al.*^28^ investigated the adsorption and inhibitory efficiency of acrylonitrile derivatives by means of a range of electrochemical methods. These techniques included: (*E*)-2(3-carbonyl 1-indole-3-)-3-(2-oxo-2-phenylethylthio)3*H*-indole-3carbonyl-3-(phenylamino)acrylonitrile (a), ((*E*)-2-*b*) and (*Z*)-3-mercapto-3-(phenylamino)acrylonitrile-2-(carbonyl-3-indole)-3 phenyl acrylonitrile (c) in 0.5 M HCl on the Cu10Ni alloy. The percentage of IE was 95.1%, 92.1%, and 90.4% for (a), (b), and (c), in that order. The effects of “3-methyl 4-amino 1,2,4 triazole on the oxidization behavior of Cu–Ni 70–30 (Monel) in a 3% NaCl solution at 25 °C are evaluated by Deyab *et al.*^29^ At 60 ppm, the inhibitor (MATA) reached its maximum percentage of IE (95%) and the inhibitor's efficacy decreased with increasing dose. Aesculus Hippocastanum Seeds (AHS) extract was employed by Loto *et al.*^30^ as a corrosion inhibitor to lower the danger of copper alloy corrosion during the acid cleaning (5 N H_3_PO_4_) process in desalination facilities. The findings indicated that the AHS extract has a 96.1% inhibitory efficiency as a corrosion inhibitor at 100 ppm. The study conducted by Li *et al.*^31^ examined the effects of admixing 1 : 1 “*Citrus paradisi* and Cymbopogon distillates on Cu–Ni alloy in 0.25 M H_2_SO_4_–3.5% NaCl electrolyte”. The results showed that the Cu–Ni alloy degradation rate without distillate is 0.373 mm per year, while at 1% dose, the degradation rate decreased to 0.140 mm per year (1.37 × 10^−5^ A cm^−2^), indicating an inhibition efficiency of 62.46%. Inhibition efficiency varied from 80.47 to 95.58% at a dosage of 2–5 percent. In a 3.5% sodium chloride solution, the corrosion inhibition behavior of methionine (Met), an environmentally beneficial amino acid, and its synergistic effect with sodium silicate (SS) for copper–nickel alloy (Cu–10Ni) were studied.^32^ According to the findings, Met has the best inhibitive efficacy of any cathode corrosion inhibitor, measuring 62% at an additional concentration of 40 ppm. Due to the synergistic action of two corrosion inhibitors, a compact and uniform layer forms on the surface of the cupronickel specimen when 20 ppm met and 20 ppm SS are added concurrently, increasing the inhibitive efficiency to 90%. In a corrosive solution containing 6% HCl with ethylenediamine (EDA) and tetraethylenepentamine (TEPA) as corrosion inhibitors, Mohamed *et al.*^33^ investigate the effects of temperature and inhibitor dosage on the corrosion of a copper-nickel alloy. At 40 °C, the highest % IE for TEPA and EDA was determined to be 56.3% and 77%, respectively. The efficiency of inhibition increases with an increase in inhibitor dose and declines with an increase in temperature. Green tea aqueous extract is used as a durable corrosion inhibitor for two Cu–Ni alloys in 3.5% NaCl, according to Gaber *et al.*^34^ The capabilities of profitable freshening water (lime water) were compared with this one. The results show that the extract under test was able to effectively slow down the rate at which alloys rusted in 3.5% NaCl. In sulfide-polluted salt water, Cu10Ni alloys electrochemical and SCC characteristics are investigated by Abd El-Nazeer *et al.*^35^ Additionally, they evaluate how glycine (gly) affects the Cu10Ni alloy's susceptibility to SCC in these media and investigate any possible synergistic effects between gly and KI on the effectiveness of erosion inhibition. The maximum efficiency is 87.32% in the presence of KI and 80.54% in the free KI solution.

This paper aims to assess the inhibitory effect of LSE on Cu–Ni alloys corrosion in a 3.5% NaCl solution. WL, PDP, EIS, EFM, and surface morphology experiments were used to confirm the LSE corrosive inhibition. Data on kinetics and thermodynamics were also assessed.

## 2. Experimental

### 2.1 Materials and solutions

The chemical makeup (wt%) of the two kinds of copper-nickel alloys employed in this investigation is as follows: 1.67 Fe, 0.768 Mn, 9.27 Ni, and a balance of Cu were found in the Cu–10Ni alloy, whereas 0.02 Fe, 0.44 Si, 0.51 Ca, 29.16 Ni, and a balance of Cu were found in the Cu–30Ni alloy. “Prior to each experiment, the working electrode was polished with SiC, rinsed with ethanol and finally with doubly distilled water”. “The auxiliary electrode was platinum wire, while a reference electrode calomel electrode (SCE), connected to a conventional electrolytic cell of capacity 100 mL”. The experiments were studied the corrosion behavior of two of Cu–Ni alloys in 3.5% NaCl solution in the absence and presence of different dose of LSE as a natural product (50, 100, 200, and 300 ppm). “All solutions were freshly prepared using analytical grade reagents and doubly distilled water”. The corrosion inhibition of Cu–Ni alloys in 3.5% NaCl solution, which has a salt content similar to seawater, was assessed using chemical and electrochemical methods in the absence and presence of various dosages of LSE as a naturally occurring product.

### 2.2 Preparation of plant extracts

**Scheme 1:**
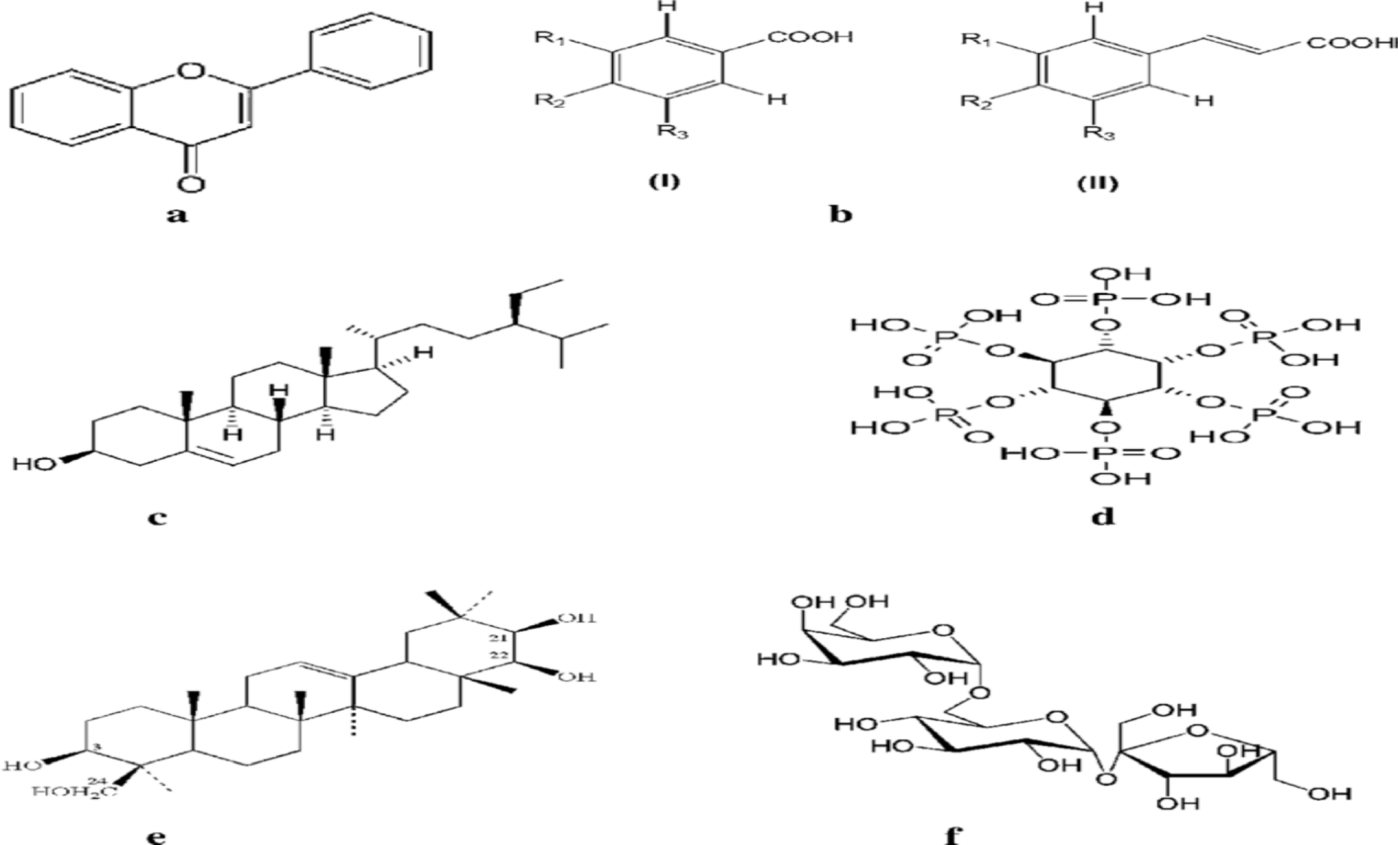
Chemical structures of lentils extract (a–f).

Different parts of the selected plants were collected from various regions of Daqahlia Governorate January 2023. These included leaves, bark and roots of lentils. The plant was identified at the Botanical herbarium at Sadat City University. Each plant sample was air dried and ground into a fine powder by used an electrical mill. Methanol is known to be powerful extraction solvent of compounds from plants.^36^ Therefore, two hundred grams (200 g) of this powder was soaked in 800 mL of methanol, at a ratio of 1 : 4 (powder/solvent) for 48 h at room temperature. Under vacuum the extract was removed and dried by rotary evaporator.^37^ The extract was then concentrated at 68 °C under reduced pressure to obtain the residues that constituted the crude extract. The investigated extract was liquefied in ethanol (1 g L^−1^) and conserve into refrigerator. All of the extracts were kept at 4 °C until further use. The main chemical structure existing in lentils extract before and after study of the corrosion inhibition are represented below (Scheme 1).

### 2.3 Weight loss (WL) measurements

The WL approach is the simplest way to evaluate corrosion losses. The samples are first properly cleaned, after which they are polished with different grades of SiC, washed in ethanol, rinsed in doubly distilled H_2_O, and then allowed to dry. Following these stages, the specimens were precisely weighed before being submerged in 100 mL of 3.5% NaCl in both the attendance and lack of LSE natural products (50, 100, 200, and 300 ppm). At a temperature of 25 °C, WL measurements were collected over 15 days. The specimens were removed from the test solution, completely cleaned of corrosion products, and then allowed to dry before being precisely reweighed. “Triplicate samples were used to check reproducibility of results”. Eqn (1) & (2) (ref. 38) can be used to get the average WL in grams and the corrosion rate (CR), respectively:

1 Δ*W* = *W*_1_ − *W*_2_

This is how the corrosion rate (CR): is calculated2
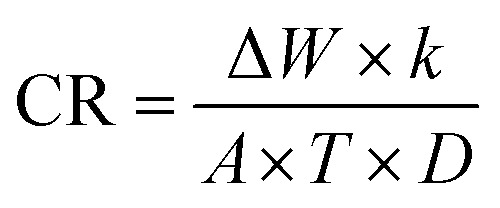
where *T* is the immersion duration in days, *A* is the Cu–Ni electrode's area in cm^2^, Δ*W* is the WL in kg, *D* is the electrode's density in grams per cube centimeter, and *k* is a constant (8.76 × 10^4^). Eqn (3) yields the inhibition efficiency (% IE) and surface coverage (*θ*).3
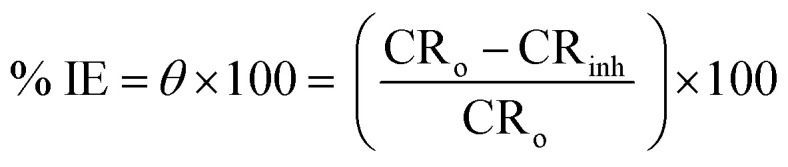
where CR_o_ and CR_inh_ are the corrosion rates of the alloys in the absence and existence of LSE, respectively.

### 2.4 Electrochemical tests

#### 2.4.1 Potentiodynamic polarization (PDP) tests

“PDP of the two Cu–Ni alloys were carried out at range of potential from (−800 to 300 mV) and scan rate 0.2 mV s^−1^” in all solutions with and without LSE, then Tafel lines are plotted and the CR without (CR_o_) and with (CR_inh_) extract, corrosion potential (*E*_corr_) values were recorded. The % IE and *θ* are determined by eqn (4).

4 % IE = [1 − (*i*_corr(inh)_)/(*i*_corr(free)_)] × 100 = *θ* ×100

where *i*_corr(inh)_ and (*i*_corr(free)_) are the corrosion current density in presence and absence of the extract, respectively.

#### 2.4.2 EIS tests

Using ac signals at open circuit potential, EIS of Cu–Ni alloys was performed in the frequency range of 100 kHz to 10 mHz with an amplitude of 10 mV peak-to-peak. The following eqn (5) defines the *θ* and the percentage IE that were received from the EIS^38^5
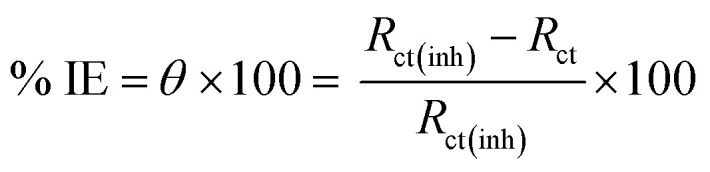
where *R*_ct_ and *R*_ct(inh)_ are the charge transfer resistance in the absence and presence of LSE, respectively. The main parameters deduced from the analysis of Nyquist diagram are the resistance of charge transfer *R*_ct_ (diameter of high frequency loop) and the capacitance of double layer *C*_dl_. Since the electrochemical theory assumed that 1/*R*_ct_ is directly proportional to the capacity of double layer.

### 2.5 Adsorption isotherm

The type of adsorption isotherm can reveal more details about the characteristics of the LSE under study. To get the adsorption isotherm, the extent of surface coverage (*θ*) of the two Cu–Ni alloys by the LSE must be evaluated by diverse adsorption isotherms. In this investigation, the (*θ*) for altered doses of the LSE in marine solution has been estimated from the WL, PDP, and EIS methods.

### 2.6 Spectroscopic analysis

We looked into the surface morphology of the two Cu–Ni alloys with and without LSE. The Cu–Ni alloys were polished and honed before being dipped into the electrolyte solutions. Following a 15-days immersion in NaCl solutions, SEM micrographs of the alloy's surface were acquired, both with and without the LSE under examination. In this work, the JEOL JSM model scanning electron microscope (SEM) at Mansoura University in Egypt was coupled with an energy dispersive spectroscopy (EDX).

## 3. Results and discussion

### 3.1 Impact of LSE as corrosion inhibitor

**Fig. 1:**
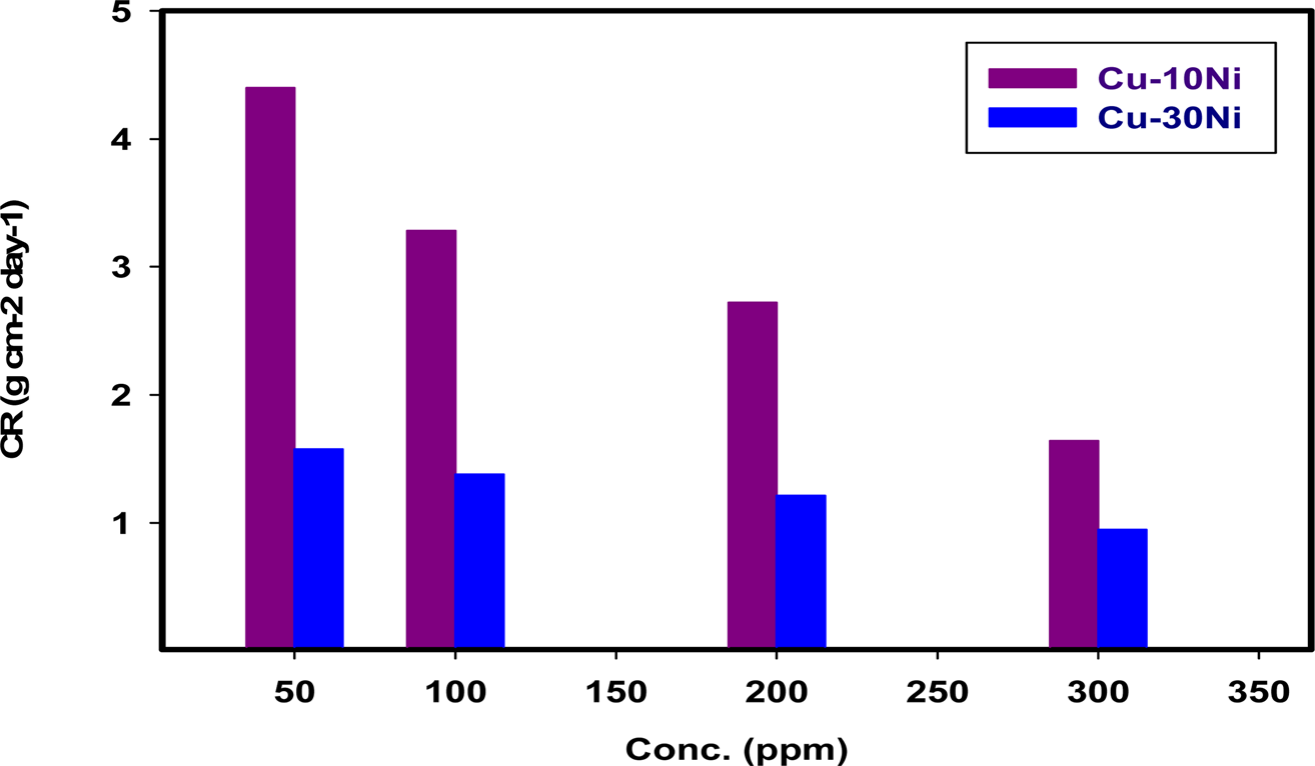
The variation of dissolution rate on two “Cu–Ni alloys” in 3.5% NaCl with different doses of LSE at 298 K after 15 days immersion.

% IE and CR values for “two Cu–Ni alloys” achieved by the method of WL at different doses of LSE are condensed in Table 1 and Fig. 1. As seen in presence of extract, the WL of the two alloys decreased. The CR becomes lower with increasing the dose of LSE. This action may be attributed to a competitive step, including passive film healing by the extract and passive film damage by the aggressive ions. The outcomes revealed that extract hinder the consumption of Cu–Ni alloys. % IE increments pointedly with increments in extract dose arriving at the most extreme estimation of 86.7% in presence of 300 ppm of extract for Cu–10Ni alloy and 73.5% for Cu–30Ni alloy. This conduct could be clarified by the adsorption of segments of the extract on the surface of Cu–Ni alloys bringing about the hinder of the dissolution and protecting the surface in the corrosive medium. By increasing the dose of the extract in the corrosive solution, the effectiveness of inhibition was improved. “As a result, a protective film that is more durable and sticky forms on the surface”.^39^ Thusly, we can infer that the LSE is a good inhibitor for Cu–Ni alloys in 3.5% NaCl solution.

**Table 1:** % IE, CR, and *θ* for “Cu–Ni alloys in 3.5% NaCl solution” with and without different doses of LSEs following 15 days of immersion

Conc. ppm	Cu–10Ni alloy			Cu–30Ni alloy		
	CR (g per cm^2^ per day)	Coverage surface, (*θ*)	% IE	CR (g per cm^2^ per day)	Coverage surface, (*θ*)	% IE
3.5% NaCl (blank)	12.4017	—	—	3.5762	—	—
50	4.3964	0.645	64.5	1.5748	0.559	55.9
100	3.2808	0.735	73.5	1.3779	0.615	61.5
200	2.7231	0.780	78.0	1.2139	0.661	66.1
300	1.6404	0.867	86.7	0.9482	0.735	73.5

### 3.2. Effect of immersion time

**Fig. 2:**
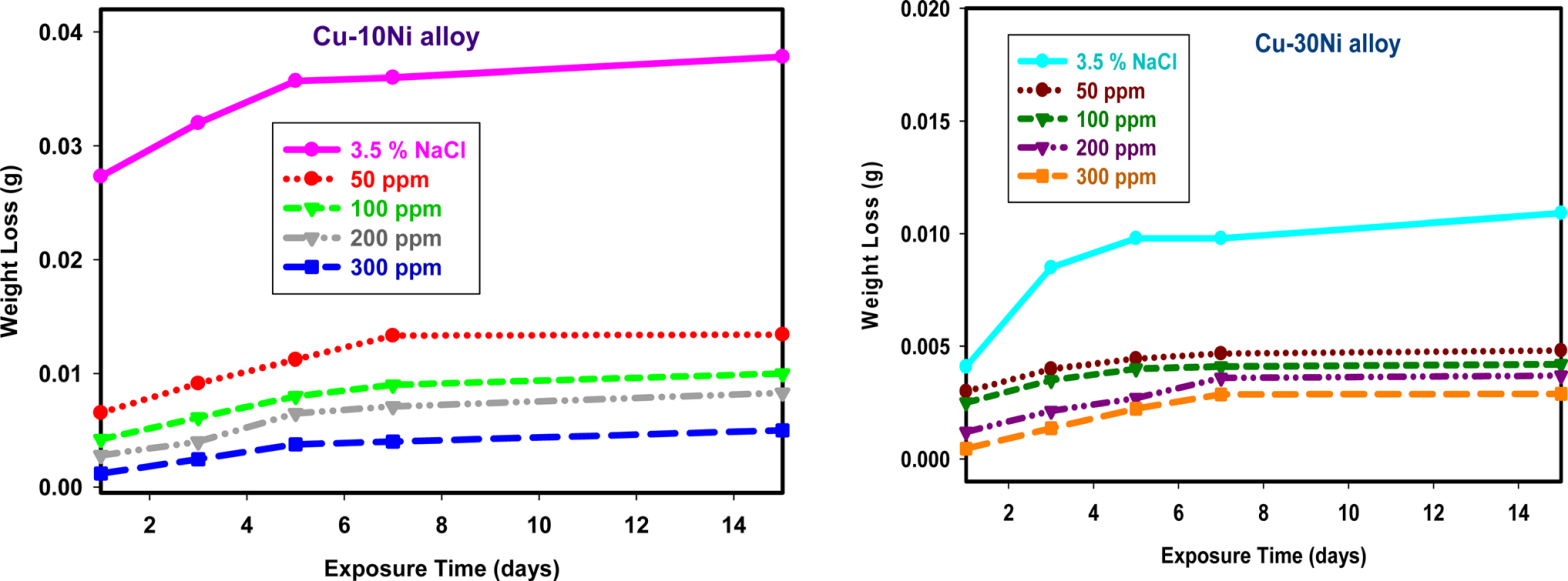
WL-time curves for used alloys dipped in 3.5% NaCl with and without different doses of LSE.

One crucial factor that can be used to assess the stability of inhibitor function is the immersion time.^40^Fig. 2 shows how the WL of Cu–Ni alloys in 3.5% NaCl changes over time, both with and without varying the dose of LSE. The graph indicates that WL rises as the duration of contact time does.

The dissolution rate of used alloys in 3.5% NaCl^41^ reduces as the dose of LSE increases, suggesting that LSE slows down the rate of corrosion. The phytochemical ingredients included in LSE are responsible for the extract's inhibitory impact. The WL of Cu–Ni alloys was shown to rise with temperature at higher temperatures (plots not shown), indicating desorption of the adsorbed protective layer from the alloy surface at higher temperatures.

### 3.3 PDP measurements

**Fig. 3:**
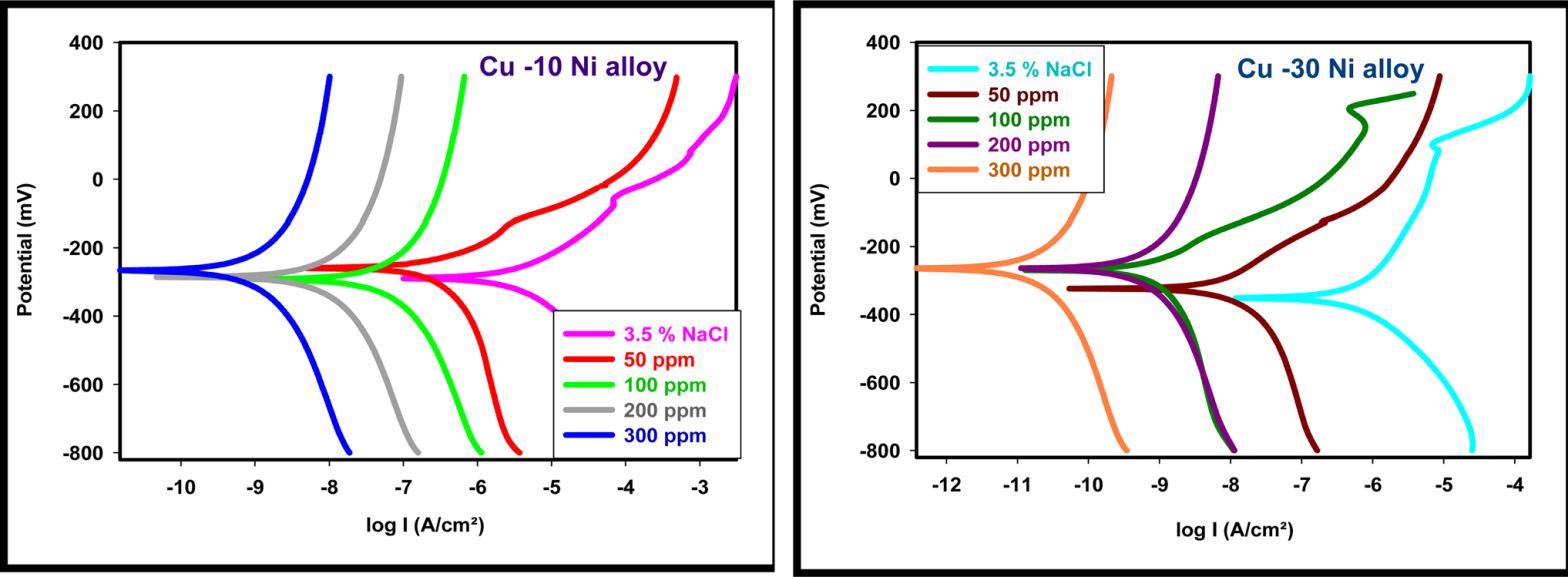
PDP curves for used alloys in 3.5% NaCl with and without altered doses of LSE.

The PDP study offers important information about the kinetics of the processes that take place at the anodic and cathodic regions as well as the kind of inhibition that corrosion inhibitors use based on corrosion potential. Fig. 3 displays the PDP curves for the two used alloys in a 3.5% NaCl solution with and without varying percentages of LSE. This figure makes it evident that the presence of LSE increased the anodic and cathodic overvoltage and caused a slight displacement in the Tafel lines, indicating that the extract acts as a mixed-type inhibitor by affecting the retardation of the two reactions (cathodic hydrogen reduction and anodic metal dissolution) without altering the dissolution mechanism.^42^Table 2 provide the electrochemical properties of the used alloys, including a percentage of IE and CR, “as well as corrosion potential, *E*_corr_, anodic Tafel slope *β*_a_, cathodic Tafel slope *β*_c_, and corrosion current density, *I*_corr_”. It is evident from these tables that *I*_corr_ values fell as LSE dosages increased. Cang *et al.*^43^ discovered similar behavior. According to Table 2, there is a shift of <85 mV (20.4 to 25.9 mV) in the *E*_corr_ values of inhibited systems compared to the uninhibited solution. The extract's ability to inhibit alloy oxidation and reduce hydrogen evolution is indicated by the tendency of *E*_corr_ values to move in a positive direction. This suggests that the extract functions as a mixed-type corrosion inhibitor.

**Table 2:** Corrosion parameters for used alloys in 3.5% NaCl solution with and without different doses of LSE

	Conc., ppm	−*E*_corr_, mV *vs.* SCE	*I*_corr_, μA cm^−2^	*β*_a_, mV dec^−1^	−*β*_c_, mV dec^−1^	CR μm per year	% IE
Cu–10Ni alloy	3.5% NaCl (blank)	298.6	35.830	97.6	209.0	419.0	—
	50	295.7	3.283	111.9	112.7	26.70	90.84
	100	292.6	1.972	40.9	59.5	23.06	94.49
	200	291.7	1.551	158.0	156.7	6.451	95.67
	300	272.7	0.807	195.6	193.4	5.940	97.74
Cu–30Ni alloy	3.5% NaCl (blank)	389.3	25.378	122.3	157.1	296.8	—
	50	379.7	3.166	116.6	110.0	37.02	91.47
	100	275.9	0.886	99.1	170.6	6.853	96.51
	200	269.9	0.531	191.7	190.9	6.210	97.90
	300	268.9	0.271	131.1	133.5	4.342	98.93

### 3.4 EIS technique

**Fig. 4:**
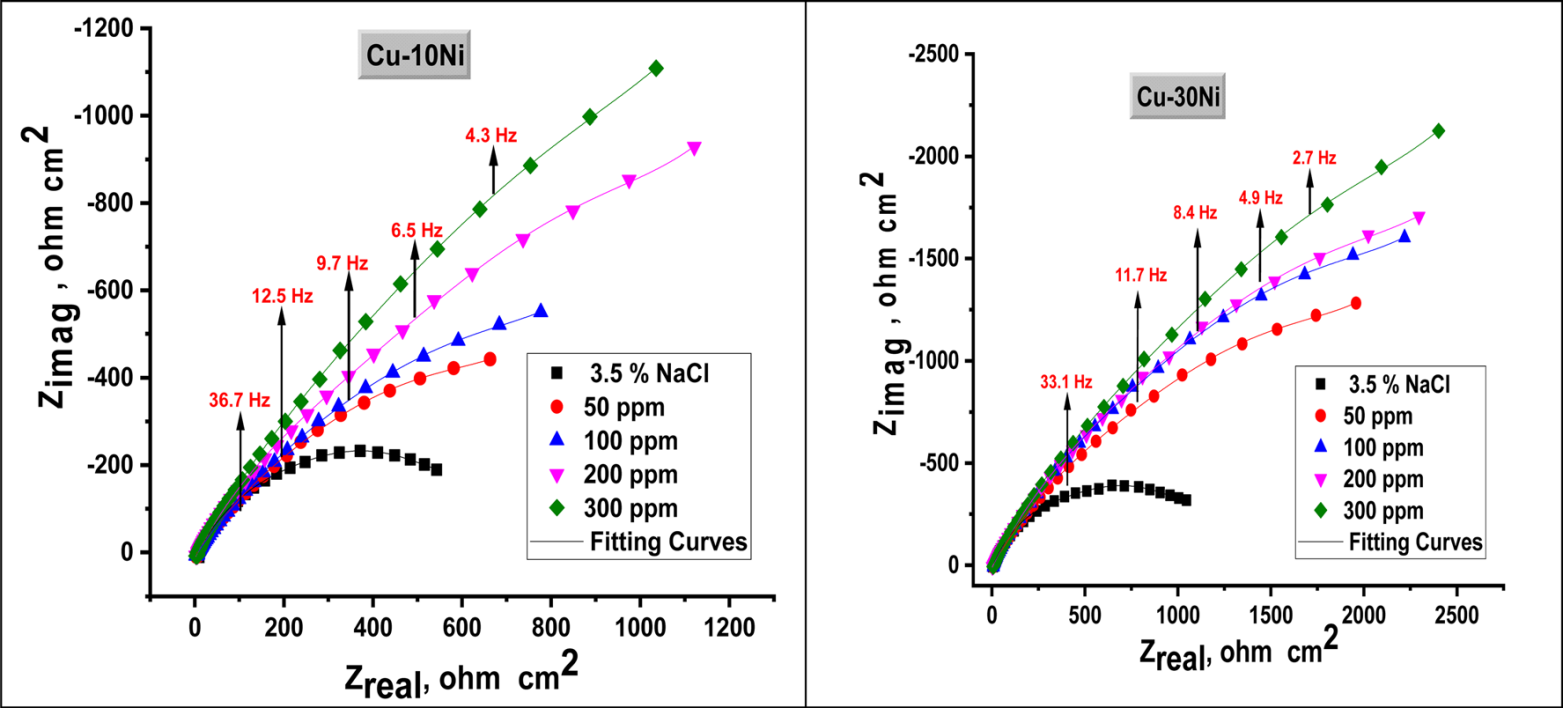
Nyquist plot for used alloys in 3.5% NaCl solution in the absence and presence of different doses of LSE at 25 °C.

EIS studies of the Cu–Ni alloys in the presence and absence of various dosages of LSE were conducted in order to learn more about the inhibitor process and to validate the results of the WL and PDP experiments. The Nyquist curve for the Cu–Ni alloys in a 3.5% NaCl solution, both in the absence and with varying dosages of LSE, is displayed in Fig. 4. Table 3 lists the EIS parameters, capacitance double layer *C*_dl_, charge transfer resistance *R*_ct_, the *θ*, and the percentage of IE. A depressed semicircle with the center under the real axis can be seen in the impedance spectra of Cu–Ni alloys in 3.5% NaCl. This type of behavior is typical of solid electrodes and is sometimes referred to as the frequency dispersion of interfacial EIS. The primary causes of this capacitance dispersion at the metal surface are the extract molecules' adsorption–desorption process on the surface of used alloys, surface roughness, and surface chemical heterogeneity.^44^ In order to address this, the equal circuit is modified to substitute a continual phase element (CPE) for *C*_dl_. CPEs are associated with surface roughness, contaminants, degree of polycrystallinity, chemical inhomogeneities, and adsorption inhibitive molecules.^45^ This is the impedance function, or CPE:^44^

6 *Z*_CPE_ = 1/*Y*_0_(*jω*)^*n*^

where *n* is the CPE exponent, which indicates the departure from the ideal performance and runs from 0 to 1, *Y*_0_ is the CPE constant, *j* is the imaginary unit, and *ω* is the angular frequency. The reduced value of *n* (Table 3) for Cu–Ni alloys in the investigated corrosive solution denotes surface irregularities brought on by corrosion-induced surface roughening of the alloys. The adsorption of extract molecules on the metal surface decreases its electrical capacity because they displace the water molecules and other ions originally adsorbed on the metal surface. This modification results in an increase of charge transfer resistance *R*_ct_ which “suggest the formation of a protective layer on the Cu–Ni alloys surface”. This layer makes a barrier for mass and charge transfer. “The data in Table 3 show that the *R*_ct_ increases with the increase in the extract dose in 3.5% NaCl solution, indicating a marked anticorrosion effect of the LSE on Cu–Ni dissolution in 3.5% NaCl solution”. The reciprocal of *R*_ct_, is directly proportional to the corrosion current density and corrosion rate.^46^ This means that the corrosion rate decreases with increasing the extract dose. “Also, the data in Table 3, show that the *C*_dl_ and *Y*_0_ values were almost decreased with the increase of the dose of investigated extract in 3.5% NaCl solution”. “*Y*_0_ for the blank is estimated to be higher than *Y*_0_ for the inhibited solution; this suggests that the extract molecules interacted with the electrode surface, minimizing the erosion of the electrode's exposed locations”.

**Table 3:** EIS parameters for used alloys in 3.5% NaCl solution in the absence and presence of altered doses of LSE at 25 °C

Alloys	Conc., ppm	*Y*_0_, μΩ^−1^, *s*^*n*^ cm^−2^	*n*	*R*_ct_, Ω cm^2^	*C*_dl_, μF cm^2^	*θ*	IE_EIS_/%	Goodness of fit (*χ*^2^)
Cu–10Ni	Blank	770	0.992	540	765	—	—	19.67 × 10^−3^
	50	732	0.979	1031	728	0.476	47.6	21.32 × 10^−3^
	100	714	0.971	1556	716	0.653	65.3	20.47 × 10^−3^
	200	688	0.969	2470	699	0.781	78.1	16.57 × 10^−3^
	300	661	0.964	2984	678	0.819	81.9	17.57 × 10^−3^
Cu–30Ni	Blank	772	0.981	1033	769	—	—	19.54 × 10^−3^
	50	712	0.976	3093	726	0.666	66.6	14.57 × 10^−3^
	100	685	0.972	3811	704	0.729	72.9	17.12 × 10^−3^
	200	679	0.970	4517	702	0.771	77.1	19.24 × 10^−3^
	300	669	0.968	6208	701	0.834	83.4	20.47 × 10^−3^

**Fig. 5:**
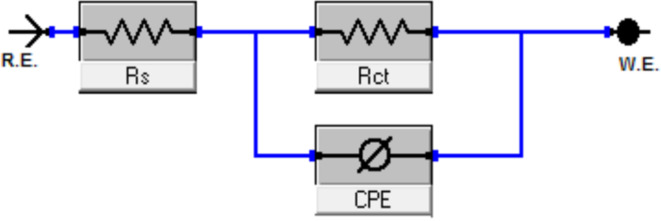
Electrical circuit model used to fit EIS results.

The decrease in *C*_dl_, which can result from a decrease in local dielectric constant and/or an increase in the thickness of the electrical double layer, suggests that LSE act by adsorption on the alloy/solution interface. The fitted results show excellent agreement with the experimental data, as indicated by the small chi-squared values (Table 3) obtained for each result. The accuracy of the fitting findings was evaluated using the chi-squared. Fig. 5 depicts the equivalent circuit that was utilized to duplicate the EIS data. Since this circuit uses CPE rather than capacitors to provide multiple forms of non-homogeneity, such as corrosive electrode surface roughness, grain boundaries, and alloy surface imperfections, we choose to employ it. The % IE calculated from EIS data are closed to those obtained from WL and PDP. The results showed the good agreement between measurements obtained from all techniques. In all measurements the % IE of Cu–30Ni alloy is greater than its in Cu–10Ni alloy at all doses of LSE, this is due to the increase of Ni content of Cu–Ni alloys in chloride solutions improve the corrosion resistance of these alloys^47^

### 3.5. Adsorption isotherms

Using eqn (3) & (4), the surface coverage (*θ*) and inhibitory efficiency (% IE) are determined based on the measurements of WL, PDP, and EIS. A number of adsorption isotherms, including Langmuir, Temkin, and Freundlich, were fitted to the data in order to analyze them.

#### 3.5.1 Langmuir adsorption isotherm

Using an appropriate fitting of the adsorption isotherm, the values of (*θ*) were graphically calculated from various techniques to examine the extract's adsorption process on the surface of Cu–Ni alloys. Using the following equation, the best connection was derived from the Langmuir adsorption isotherm:^48^7
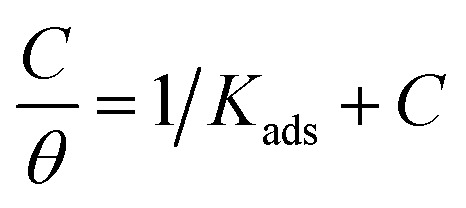


**Fig. 6:**
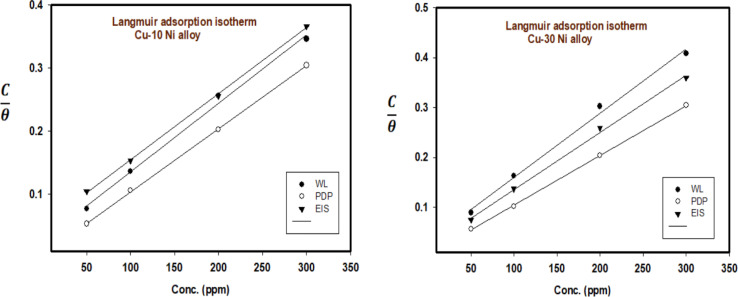
Langmuir isotherm plot for the corrosion of Cu–Ni alloys in 3.5% NaCl solution in the absence and presence of different doses of LSE at 25 °C.

The inhibitor adsorption equilibrium constant, *K*_ads_, is defined as follows: *C* represents the inhibitor dose. A plot matching the Langmuir adsorption isotherm is shown in Fig. 6, with *C*/*θ* as the *X*-axis and *C* as the *Y*-axis. For Cu–10Ni and Cu30Ni alloys, respectively, a perfect linear curve was generated with regression constants *R*^2^ > 0.9982 and > 0.9973. The equilibrium constant of the inhibitor's adsorption is often related to Δ*G*_ads_. It might be calculated using eqn (8):^48^

8 *K*_ads_ = 1/55.5 exp(−(Δ*G*_ads_/*RT*))

where 55.5 was the molar dose value of water in solution in mol dm^−3^, *R* is the gas constant, *T* is the absolute temperature, and Δ*G*_ads_ is defined as the standard free energy of inhibitor adsorption. Table 4 presents the calculated standard Gibbs free energy of adsorption of the extract at 298 K. When the values of Δ*G*_ads_ are around −20 kJ mol^−1^ or lower, the adsorption is due to the electrostatic interaction between the charged molecules of the extract and the charged electrode (physisorption). Meanwhile, those more negative than −40 kJ mol^−1^ reveal the charge transfer from the extract to the surface of the alloys to form a coordination bond (chemisorption)^49^ In this current work, the obtained negative values of Δ*G*_ads_ (less negative than −20 kJ mol^−1^) show that the adsorption process of the extract on Cu–Ni alloys in 3.5% NaCl solution is spontaneous, also indicates the stability of adsorbed layer on Cu–Ni alloys surface and the adsorption of the studied LSE follows physisorption. The Temkin and Freundlich isotherms data and figures are represented in Fig 1S and 2S.†

**Table 4:** Collects the adsorption parameters for all mentioned adsorption isotherms that obtained from graphical representation

Isotherms	Corrosion tech	Cu–10Ni alloy			Cu–30Ni alloy		
		*K*_ads_	*R* ^2^	−Δ*G*_ads_	*K*_ads_	*R* ^2^	−Δ*G*_ads_
Langmuir	WL	35.907	0.9952	18.822	30.7756	0.9947	18.441
	PDP	0.231	0.9999	6.322	0.18764	0.9997	5.805
	EIS	19.952	0.9994	17.367	45.4317	0.9975	19.406
Temkin	WL	0.133	0.9627	4.944	0.04473	0.9570	2.253
	PDP	1.048	0.9097	10.067	0.71254	0.7252	9.111
	EIS	0.229	0.9755	6.296	0.19386	0.9737	5.886
Freundlich	WL	0.347	0.9691	7.328	0.30073	0.9705	6.974
	PDP	1.066	0.9111	3.878	0.82164	0.7221	9.464
	EIS	0.086	0.9519	3.878	0.42217	0.9798	7.814

### 3.6 Impact of temperature on inhibition process

**Fig. 7:**
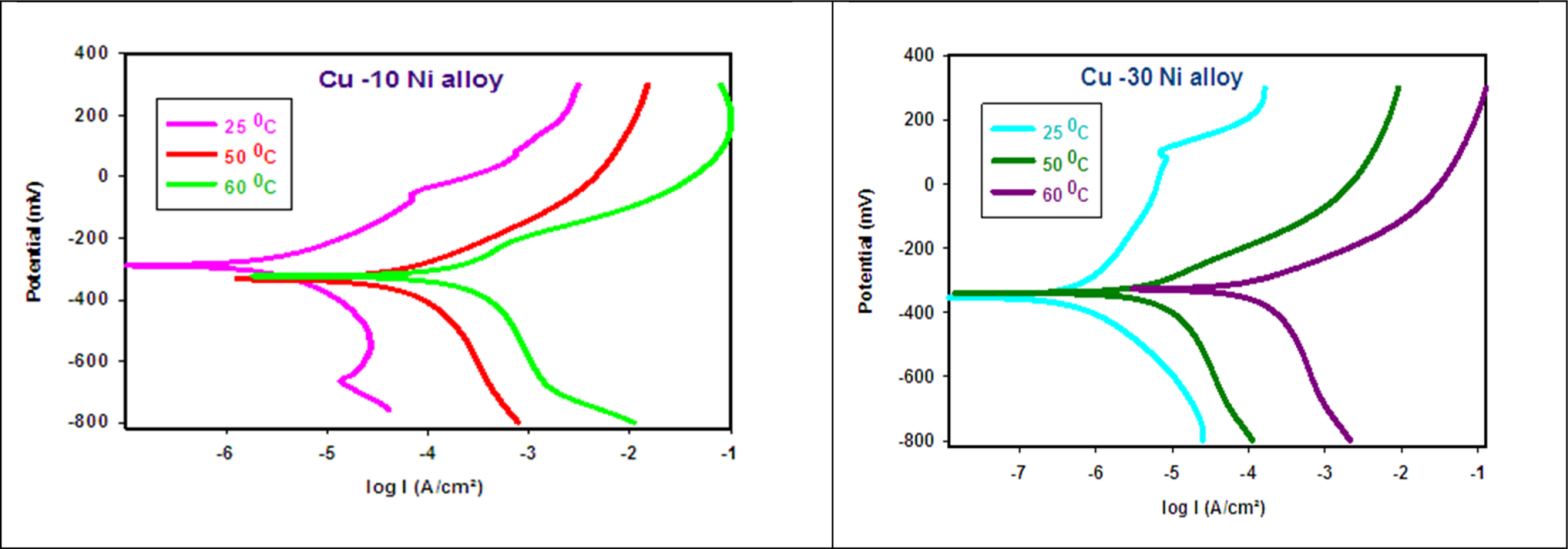
PDP curves for Cu–Ni alloys in 3.5% NaCl solution at different temperatures.

**Fig. 8:**
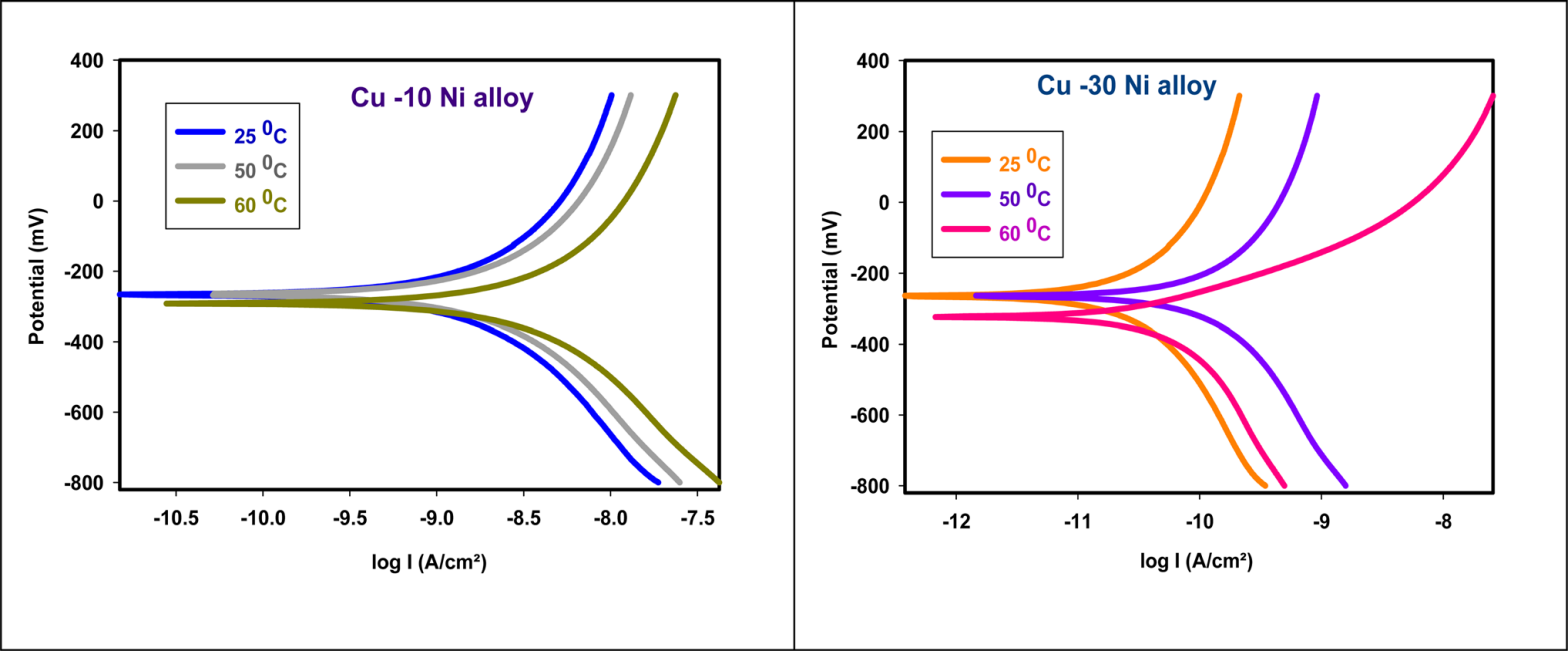
PDP plots for used alloys in 3.5% NaCl solution in attendance of 300 ppm LSE at altered temperatures.

**Fig. 9:**
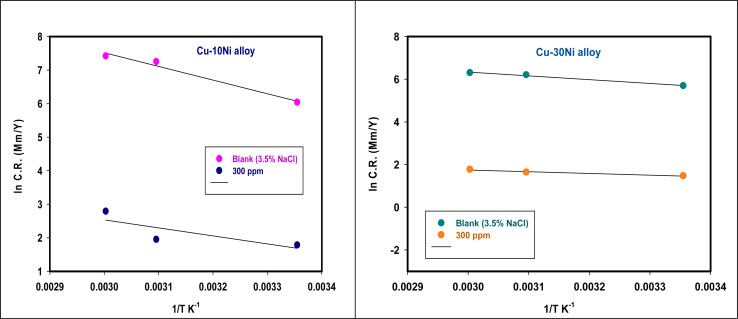
A plot of Ln CR *versus* 1/*T* for Cu–Ni alloys in 3.5% NaCl containing 300 ppm LSE altered temperatures.

By using PDP tests the effect of temperature on used alloys in 3.5% NaCl solution in lack and attendance of 300 ppm LSE was studied in the temperature extent (25–60 °C). The CR, *I*_corr_, *E*_corr_ and Tafel constants of Cu–Ni alloys in 3.5% NaCl solution as a function of temperature in absence presence of 300 ppm of LSE are summarized in Tables 5 & 6, respectively. To compute activation thermodynamic functions of the corrosion process, Arrhenius eqn (11) was utilized.^50–52^11
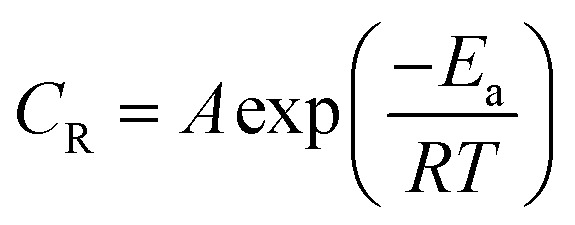
where 
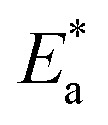
 is the activation energy, *R* is the gas constant, *A* is the pre-exponential factor of Arrhenius. Fig. 7 & 8 show the PDP curves for two used alloys in 3.5% NaCl solution in the absence and presence of 300 ppm LSE at altered temperatures, respectively. The electrochemical parameters such as *E*_corr_, *I*_corr_, anodic (*β*_a_) and cathodic (*β*_c_) Tafel slopes, and CR are tabulated in Tables 5 and 6 It is clear from the Table 5 that, *I*_corr_ decreases with increasing doses of LSE and hence % IE increases, on other hand, in Table 6*I*_corr_, for used alloys, increases with increasing temperature, and hence lowering in % IE. Fig. 9 represents the Arrhenius plots of the Ln CR *vs.* 1/*T* for the used alloys in 3.5% NaCl with and without 300 ppm LSE at various temperatures. From the slopes of these plots – 
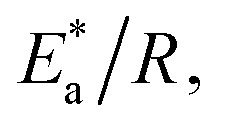
 the activation energy 
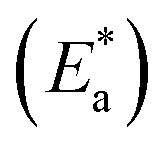
 values were calculated. The calculated 
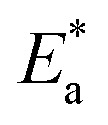
 values and regression coefficient of Cu–10Ni alloy in 3.5% NaCl with 300 ppm LSE were 34.47 kJ mol^−1^ and 19.83 kJ mol^−1^, 0.9458, and 0.9653, respectively, while for the Cu–30Ni alloy were 14.43 kJ mol^−1^, 6.77 kJ mol^−1^, and 0.9790, 9653, respectively. Arrhenius indicates that the faster the dependence of the CR with the temperature the higher 
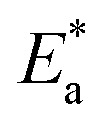
.^53^ “It is noticed that the formation of adsorbed film on the surface of the alloys with increasing thickness that decrease the dissolution of used alloys”. The increase values of 
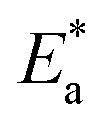
 with LSE than in its absence for the two alloys indicate the physical adsorption of LSE on alloys surface.

**Table 5:** Corrosion parameters for Cu–Ni alloys in 3.5% NaCl solution at different temperatures

	Temp., °C	−*E*_corr_, mV *vs.* SCE	−*β*_c_, mV dec^−1^	*β*_a_, mV dec^−1^	*I*_corr_, μA cm^−2^	CR, μm per year
Cu–10Ni alloy	25	292.6	209.0	97.6	35.830	419.0
	50	339.1	141.2	104.2	120.189	1405
	60	327.8	169.1	157.9	142.098	1662
Cu–30Ni alloy	25	358.3	157.1	122.3	35.378	296.8
	50	344.2	129.6	99.7	41.209	493.6
	60	333.2	88.4	62.8	46.519	544.0

**Table 6:** Corrosion parameters for used alloys in 3.5% NaCl solution in attendance of 300 ppm LSE at various temperatures

	Temp., °C	−*E*_corr_, mV *vs.* SCE	*I*_corr_, μA cm^−2^	*β*_a_, mV dec^−1^	−*β*_c_, mV dec^−1^	CR, μm per year	% IE
Cu–10Ni alloy	25	272.7	0.807	195.6	193.4	5.940	98.58
	50	272.0	1.000	189.0	182.5	7.014	94.16
	60	297.3	1.392	64.4	71.3	16.28	88.54
Cu–30Ni alloy	25	268.9	0.271	131.1	133.5	4.342	87.73
	50	264.2	0.441	100.3	152.9	5.154	87.49
	60	329.5	0.502	180.7	180.8	5.870	87.38

### 3.7 Surface analysis

#### 3.7.1 SEM/EDX investigation

**Fig. 10:**
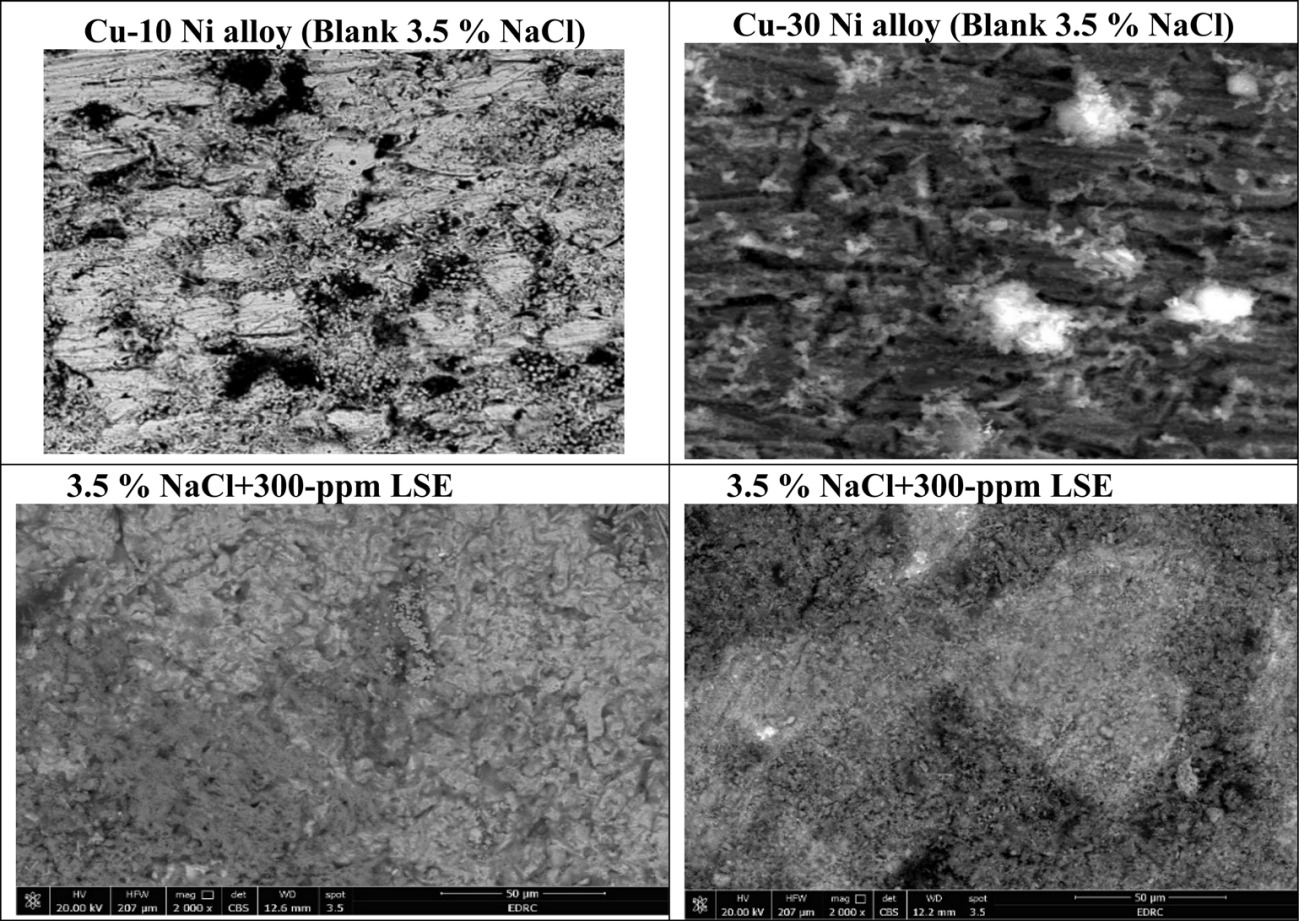
Surface morphology for Cu–Ni alloys exposed to 3.5% NaCl solution with inhibitor.

**Fig. 11:**
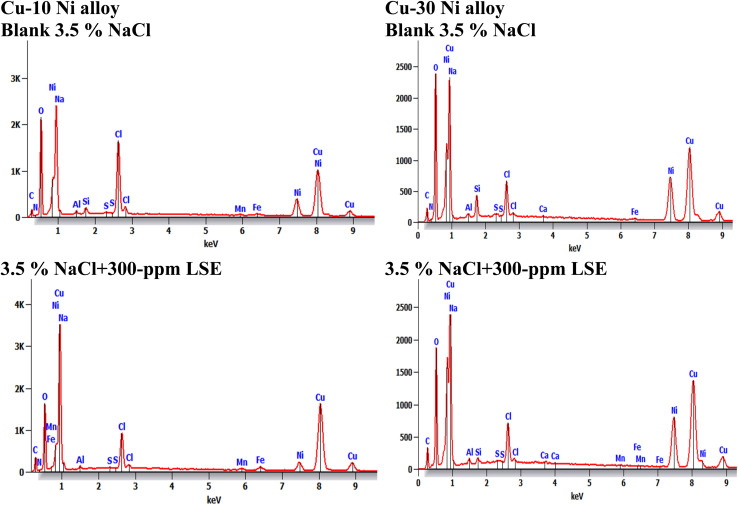
EDX spectra for the used alloys exposed to 3.5% NaCl with LSE.

Fig. 10 obviously illustrates the SEM images of Cu–Ni alloys exposed to 3.5% NaCl solution with and without LSE. “The SEM images of the corroded Cu–Ni alloys after submersing in 3.5% NaCl solution and which appeared as a rough surface with many cracks and pores”. “This result is due to the Cl^−^ ions in the medium aggressively attacking the electrode surface, causing surface corrosion”. “On the other hand, in the presence of LSE, less destruction was observed in the micrographs of the electrodes”. “These results confirm that the surface has greatly enhanced, with a valuable and observable reduction in the corrosion rate”. “This improvement is owing to the creation of a good protective layer on the used alloys surface from the LSE, which prevents the corrosion”. “EDX spectra of the two used alloys are shown” in Fig. 11. This figure showed that the elements present on the surface of Cu–Ni alloys. Table 7 depicted the EDX spectrum results of the Cu–Ni alloys specimen for elemental composition with and without extract. This reveals that the predominant species, which is found on the electrodes surface, is form an adsorbed protective layer on alloys surface. The EDX analysis of the surface in the presence of LSE showed the presence of oxygen along with the reduction of the chloride signal. This is possibly due to the adsorption of LSE *via* oxygen active centers on the Cu–Ni alloys surface”. In addition, the less porous property of the inhibitor-covered surface resulted in less penetration of the chloride ions onto the used alloys.

**Table 7:** EDX spectrum results of the Cu–Ni alloys specimen for elemental composition with and without extract

Element	Without extract (wt%) for Cu–10Ni	With LSE, Cu–10Ni alloy (wt%)	Without extract (wt%), for Cu–30Ni	With LSE, Cu–30Ni alloy (wt%)
O	17.2	10.9	16.4	14.6
Na	1.1	0.8	0.7	0.5
Cl	10.2	1.9	3.3	2.5
Mn	0.6	0.6	0.1	0.1
Fe	1.1	1.4	0.4	0.2
Ni	14.0	7.7	23.9	24.6
Cu	51.8	68.9	51.1	56.4
Si	—	—	1.7	0.7

#### 3.7.2 AFM analysis

**Fig. 12:**
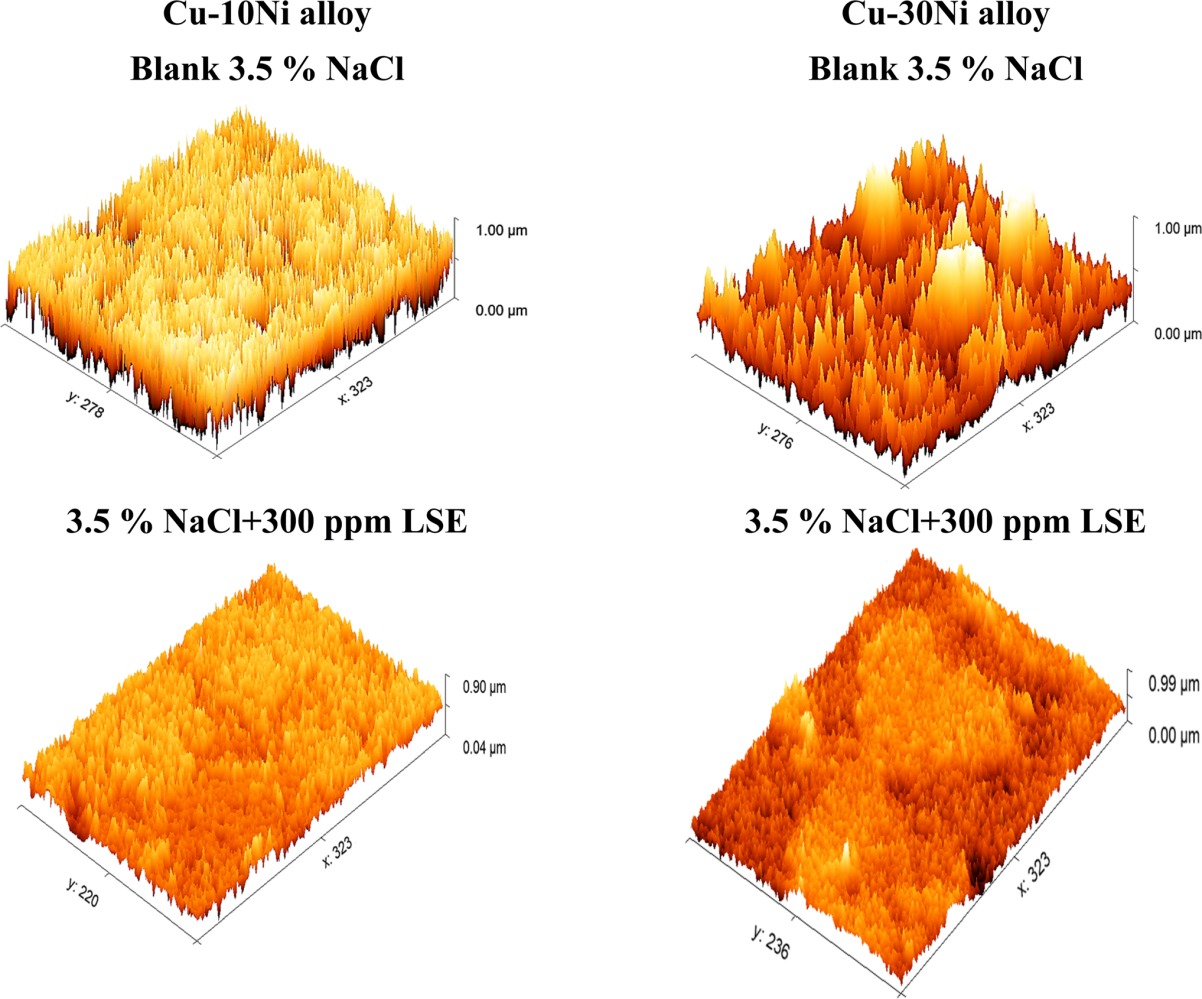
Surface morphology for Cu–Ni alloys exposed to 3.5% NaCl solution with and without LSE.

An AFM analysis was conducted on the Cu–Ni alloys surface to check the existence of an inhibitor film. AFM images and force curves are shown in Fig. 12 after 24 hours of exposure in 3.5% NaCl solution presence and absence Cu–Ni alloys corrosion inhibitors. The mean roughness value of Cu-10 Ni alloys surface that was exposed to a 3.5% NaCl solution but was not treated with the LSE was substantially greater at 300 nm and for Cu–30Ni alloy equal 400 nm. The acid's corrosive effects over the course of 3 hours rust test period left the alloys surface with a porous structure and deep fractures, which led to this heightened roughness. However, when the tested inhibitors are applied at the optimum dose (300 ppm LSE), the average roughness for Cu–10Ni alloy & Cu–30Ni alloy is reduced to 98 & 110 nm, respectively. The test LSE effectively maintain hardness of Cu–Ni alloys, as seen by the drop in roughness value.^54,55^

### 3.8 The proposed adsorption mechanism of the extract

The adsorption of an inhibitor depends on its chemical structure, its molecular size, the nature and charged surface of the metal, and distribution of charge over the whole inhibitor molecule. In fact, adsorption process can occur through the replacement of solvent molecules from the metal surface by ions and molecules accumulated near the metal/solution interface. Ions can accumulate at the metal/solution interface in excess of those required to balance the charge on the metal at the operating potential. These ions replace solvent molecules from the alloy surface, and their centers reside at the inner Helmholtz plane. This phenomenon is termed specific adsorption, contact adsorption. The anions are adsorbed when the alloy surface has an excess positive charge in an amount greater than that required to balance the charge corresponding to the applied potential. The exact nature of the interactions between the alloy surface and an aromatic molecule depends on the relative coordinating strength towards the given metal of the particular groups' present.^56^ Generally, two modes of adsorption were considered. In one mode, the neutral molecules of leaves extract can be adsorbed on the surface of alloy through the chemisorption mechanism, involving the displacement of water molecules from the alloy surface and the sharing electrons between the heteroatoms and Cu. The extract molecules can also adsorb on the alloy surface based on donor–acceptor interactions between π-electrons of the aromatic/heterocyclic ring and vacant d-orbitals of surface iron. Since all the different parts of plant extract possess several heteroatoms containing active constituents, therefore there may be a synergism between the molecules accounting for the good inhibition efficiencies.

## 4. Conclusions

From our experimental obtained values, the following conclusions can be made:

(1). In 3.5% NaCl environment, lentil seed–methanol extract showed surface active characteristics and performed well as an inhibitor to stop Cu–Ni alloy corrosion.

(2) The PDP curves indicate that this LSE behaves as mixed-type inhibitor *i.e.* LSE reduces both cathodic and anodic corrosion in NaCl solution.

(3) According to the results of the WL measurement, LSE effectively inhibits the corrosion of Cu–Ni alloys in a 3.5% NaCl solution.

(4) By increasing the temperature the % IE decreases, on the other hand, it dropped by increase the LSE dosages.

(5) The adsorption of LSE on Cu–Ni alloys surface is a physical one and obeyed, Langmuir, Temkin, and Freundlich models.

(6) There is strong correlation between outcomes derived from the WL method and PDP, EIS techniques.

## Data availability

The data that support the findings of this study are available on request from the corresponding author.

## Conflicts of interest

There are no conflicts to declare.

## Supplementary Material

RA-014-D4RA04758C-s001

## References

[cit1] Martinez S., Metikoš-Huković M. (2006). J. Appl. Electrochem..

[cit2] Badawy W. A., Ismail K. M., Fathi A. M. (2006). Electrochim. Acta.

[cit3] Badawy W. A., Ismail K. M., Fathi A. M. (2005). J. Appl. Electrochem..

[cit4] Rao B. V. A., Kumar K. C. (2017). Arabian J. Chem..

[cit5] Metikoš-Huković M., Škugor I., Grubač Z., Babić R. (2010). Electrochim. Acta.

[cit6] Rao B. V. A., Kumar K. C. (2014). J. Mater. Sci. Technol..

[cit7] Tansuğ G., Tüken T., Giray E. S., Fındıkkıran G., Sığırcık G., Demirkol O., Erbil M. (2014). Corros. Sci..

[cit8] Gelman D., Starosvetsky D., Ein-Eli Y. (2014). Corros. Sci..

[cit9] Finšgar M. (2013). Corros. Sci..

[cit10] Allam N. K., Nazeer A. A., Ashour E. A. (2010). Ind. Eng. Chem. Res..

[cit11] Allam N. K., Nazeer A. A., Ashour E. A. (2009). J. Appl. Electrochem..

[cit12] Allam N. K., Hegazy H. S., Ashour E. A. (2010). J. Electrochem. Soc..

[cit13] Allam N. K., Hegazy H. S., Ashour E. A. (2007). Int. J. Electrochem. Sci..

[cit14] Ashassi-Sorkhabi H., Asghari E. (2008). Electrochim. Acta.

[cit15] Zhang D.-Q., Cai Q.-R., He X.-M., Gao L.-X., Kim G. S. (2009). Mater. Chem. Phys..

[cit16] Fouda A. S., Ahmed R. E., El-Hossiany A. (2021). Prot. Met. Phys. Chem. Surf..

[cit17] El-Rabiee M. M., Helal N. H., Abd El-Hafez G. M., Badawy W. A. (2008). J. Alloys Compd..

[cit18] Nazeer A. A., Fouda A. S., Ashour E. A. (2011). J. Mater. Environ. Sci..

[cit19] Oguzie E. E., Li Y., Wang F. H. (2007). Electrochim. Acta.

[cit20] Butler M. S. (2004). J. Nat. Prod..

[cit21] Raja P. B., Sethuraman M. G. (2008). Mater. Lett..

[cit22] Ji G., Anjum S., Sundaram S., Prakash R. (2015). Corros. Sci..

[cit23] Bartley J., Huynh N., Bottle S. E., Flitt H., Notoya T., Schweinsberg D. P. (2003). Corros. Sci..

[cit24] Fateh A., Aliofkhazraei M., Rezvanian A. R. (2020). Arabian J. Chem..

[cit25] Mehdipour M., Ramezanzadeh B., Arman S. Y. (2015). J. Ind. Eng. Chem..

[cit26] Odewunmi N. A., Umoren S. A., Gasem Z. M. (2015). J. Environ. Chem. Eng..

[cit27] Fouda A.
S., Abdallah M., El-Hoseiny M. (2013). Afr. J. Pure Appl. Chem..

[cit28] Chieb T., Belmokre K., Benmessaoud M., Drissi S. E. H., Hajjaji N., Srhiri A. (2011). Mater. Sci. Appl..

[cit29] Deyab M. A., Mohsen Q., Guo L. (2022). J. Mol. Liq..

[cit30] Loto R. T. (2021). Chem. Data Collect..

[cit31] Li X., Li W., Yang S., Hou L. (2017). Mater. Express.

[cit32] Mahmmod A. A., Ismael M. H., Fadhil A. A., Kurshed N. H. (2019). Int. J. Corros. Scale Inhib..

[cit33] Gaber G. A., Hosny S., Mohamed L. Z. (2021). Int. J. Electrochem. Sci..

[cit34] Gaber G. A., Ghobashy M. M., Madani M., Alshangiti D. M., Alkhursani S. A., Al-Gahtany S. A., Nady N. (2021). Green Process. Synth..

[cit35] Abd El-Nazeer A., Allam N. K., Youssef G. I., Ashour E. A. (2011). Ind. Eng. Chem. Res..

[cit36] Acimovic M., Tesevic V., Todosijevic M., Djisalov J., Oljaca S. (2015). Compositional characteristics of the essential oil of Pimpinella anisum and Foeniculum vulgare grown in Serbia. Botanica Serbica.

[cit37] Wu L.-Y., Gao H.-Z., Wang X.-L., Ye J.-H., Lu J.-L., Liang Y.-R. (2010). J. Med. Plants Res..

[cit38] Gaber G. A., Aly H. A., Mohamed L. Z. (2020). Int. J. Electrochem. Sci..

[cit39] Fouda A. S., Badr S. E., Ahmed A. M., El-Hossiany A. (2021). Int. J. Corros. Scale Inhib..

[cit40] Li X., Deng S., Fu H. (2012). Corros. Sci..

[cit41] Hyba A. M., El Refay H. M., Shahen S., Gaber G. A. (2023). Chem. Pap..

[cit42] Abbas M. A., Arafa E. I., Gad E. S., Bedair M. A., El-Azabawy O. E., Al-Shafey H. I. (2022). Inorg. Chem. Commun..

[cit43] Cang H., Fei Z., Shao J., Shi W., Xu Q. (2013). Int. J. Electrochem. Sci..

[cit44] Hegazy M. A., Abdallah M., Awad M. K., Rezk M. (2014). Corros. Sci..

[cit45] Fouda A. S., Etaiw S. E. H., Ibrahim A. M., El-Hossiany A. A. (2023). RSC Adv..

[cit46] Naderi E., Jafari A. H., Ehteshamzadeh M., Hosseini M. G. (2009). Mater. Chem. Phys..

[cit47] Badawy W. A., Ismail K. M., Fathi A. M. (2005). Electrochim. Acta.

[cit48] Fouda A. S., Abd El-Ghaffar M. A., Sherif M. H., El-Habab A. T., El-Hossiany A. (2020). Prot. Met. Phys. Chem. Surf..

[cit49] Jamalizadeh E., Hosseini S. M. A., Jafari A. H. (2009). Corros.
Sci..

[cit50] Dardeer H. M., Abbas S. A., Ghobashy M. M., Gaber G. A., Aly M. F. (2023). Inorg. Chem. Commun..

[cit51] Alahiane M., Oukhrib R., Albrimi Y. A., Abou Oualid H., Bourzi H., Akbour R. A., Assabbane A., Nahlé A., Hamdani M. (2020). RSC Adv..

[cit52] Yaro A. S., Wael R. K., Khadom A. A. (2010). J. Univ. Chem. Technol. Metall..

[cit53] El-Sherif R. M., Ismail K. M., Badawy W. A. (2004). Electrochim. Acta.

[cit54] Eissa M., Etaiw S. H., El-Waseef E. E., El-Hossiany A., Fouda A. S. (2024). Sci. Rep..

[cit55] Fouda A. S., Etaiw S. E. H., Abd El-Aziz D. M., El-Hossiany A. A., Elbaz U. A. (2024). BMC Chem..

[cit56] Ritchie I. M., Bailey S., Woods R. (1999). Metal-solution interface. Adv. Colloid Interface Sci..

