# Supplementary material for: Application of lentil seed extract as an inhibitor to assess the corrosion properties of copper–nickel alloys in a NaCl environment
Source: RSC Adv. 2024 Sep 3;14(38):28044–57. doi: 10.1039/d4ra04758c (PMC11369764; doi:10.1039/d4ra04758c)
Supplement: RA-014-D4RA04758C-s001 [file RA-014-D4RA04758C-s001.pdf]

This journal is © The Royal Society of Chemistry 2024

3.4.2 "Temkin adsorption isotherm"

"The surface coverage (θ) is connected to the inhibitor dose (C) and the adsorption equilibrium constant K_{ads} as shown in" Eq. 9 [50].

$$\exp^{(-2a\,\theta)} = K_{ads} \times C \qquad (9)$$

where a is the attractive parameter and K_{ads} is the adsorption equilibrium constant. From Fig. 6 linear plots are obtained, which affirms that the adsorption obeys the Temkin adsorption isotherm. Adsorption parameters obtained from this Figure are shown in Table 4.

3.4.3 "Freundlich adsorption isotherm"

"According to the Freundlich isotherm, θ is related to the inhibitor dose C" by Eq. 10 [49].

$$\log \theta = \log K_{ads} + n \log C \qquad (10)$$

where n is the empirical constant, and the other constants have the same meaning.
Fig. 7 shows straight lines relation of log θ against log C with slope n and intercept log K_{ads}. The deduced adsorption parameters K_{ads}, n, and ΔG^0_{ads} are shown in Table 4. The obtained values of the correlation factor are far from unity. The adsorption process was studied using Langmuir, Freundlich, and Temkin isotherms. "The adsorption studies clearly indicated that the experimental data satisfied the Langmuir, Freundlich, and Temkin adsorption isotherms with good linearity"[51]. "The chosen criteria of the best-fit isotherm are based on the higher correlation coefficient". "The higher value of K_{ads} indicates that the LSE is strongly adsorbed on the Cu-Ni alloys surface". From Table 4, the fitting was obtained by Langmuir adsorption isotherm with high correlation coefficients (R) as well as with the other two models Temkin & Freundlich isotherms. "Generally, values of K_{ads} obtained from the three isotherms were in a good agreement with each other". From the results of Table 4 one can conclude that Langmuir adsorption isotherm is the most fitted one, and from its value of **-ΔG_{ads}** one can conclude that the adsorption of LSE on the alloys surface is of mixed type.

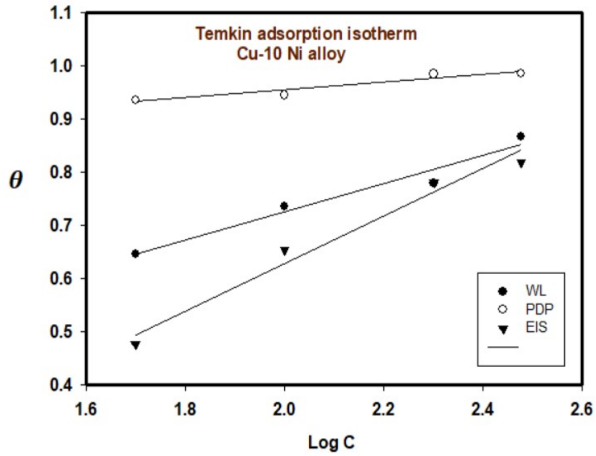

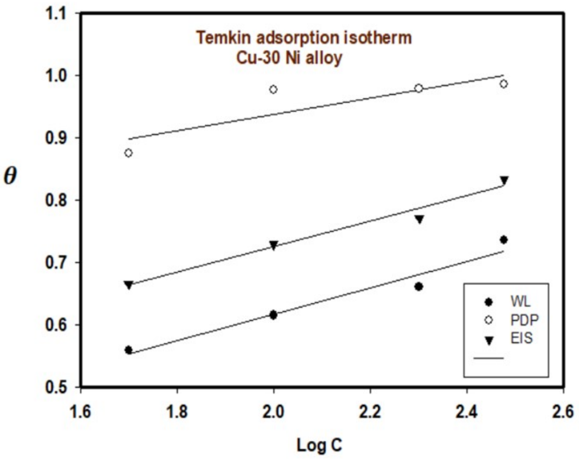

Fig. S7 Temkin isotherm plot for the corrosion of Cu-Ni alloys in 3.5 % NaCl solution in the absence and presence of different doses of LSE at 25°C

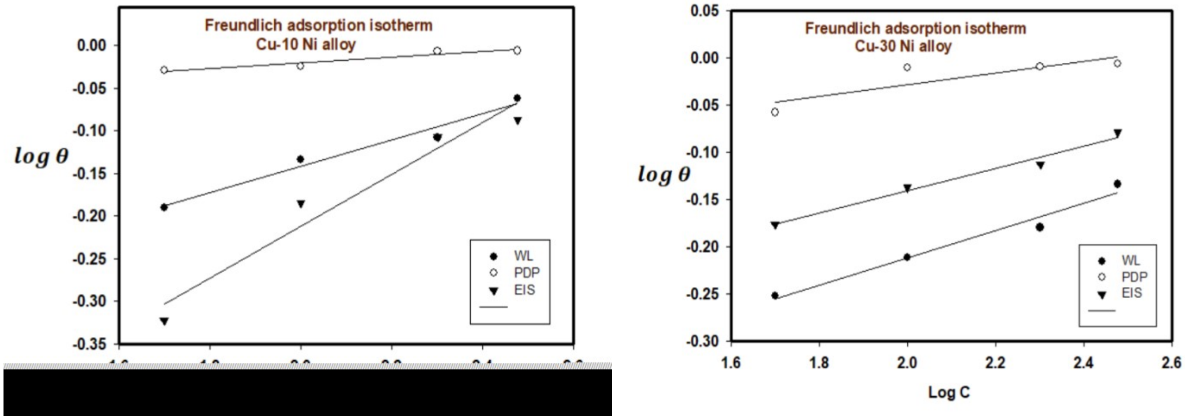

Fig. S8 Freundlich isotherm plot for the corrosion of Cu-Ni alloys in 3.5 % NaCl solution in the absence and presence of different doses of LSE at 25°C
